# Novel Object Recognition in Rats With NMDAR Dysfunction in CA1 After Stereotactic Injection of Anti-NMDAR Encephalitis Cerebrospinal Fluid

**DOI:** 10.3389/fneur.2019.00586

**Published:** 2019-06-05

**Authors:** Maxi Kersten, Theresa Rabbe, Roman Blome, Katrin Porath, Tina Sellmann, Christian G. Bien, Rüdiger Köhling, Timo Kirschstein

**Affiliations:** ^1^Oscar Langendorff Institute of Physiology, University of Rostock, Rostock, Germany; ^2^Epilepsy Center Bethel, Krankenhaus Mara, Bielefeld, Germany; ^3^Center of Transdisciplinary Neurosciences Rostock, University of Rostock, Rostock, Germany

**Keywords:** anti-NMDAR encephalitis, cerebrospinal fluid, long-term potentiation, object recognition, perirhinal cortex

## Abstract

**Purpose:** Limbic encephalitis associated with autoantibodies against N-methyl D-aspartate receptors (NMDARs) often presents with memory impairment. NMDARs are key targets for memory acquisition and retrieval, and have been mechanistically linked to its underlying process, synaptic plasticity. Clinically, memory deficits are largely compatible with a pre-dominantly hippocampus-dependent phenotype, which, in rodents, is principally involved in spatial memory. Previous studies confirmed the impaired spatial memory in the rat model of anti-NMDAR encephalitis. Here, we hypothesized that non-spatial memory functions, such as object recognition might also be affected in this model.

**Methods:** We performed stereotactic intrahippocampal bolus injection of human cerebrospinal fluid (CSF) from anti-NMDAR encephalitis and control patients into the hippocampus of the anesthetized rat. After recovery for 1–8 days, hippocampal slices were prepared from these animals and NMDAR-dependent long-term potentiation was assessed at the Schaffer collateral-CA1 synapse. In addition, we performed behavioral analyses using the open field and novel object recognition tasks.

**Results:** NMDAR-dependent long-term potentiation in the hippocampal CA1 area was significantly suppressed, indicating successful NMDAR dysfunction in this subfield. Spontaneous locomotor activity as well as anxiety-related behavior in the open field did not differ between NMDAR-CSF-treated and control animals. In the novel object recognition task, there were no differences in the motivation to approach objects. In contrast, we observed a significantly preferred exploration of the novel object only in control, but not in NMDAR-CSF-treated rats.

**Conclusion:** These results indicate that NMDAR dysfunction obtained by intrahippocampal stereotactic injection does not alter locomotor or anxiety-related behavior. In addition, approach to an object or exploratory behavior in general are not affected either, but intact initial NMDAR-dependent processes might be involved in novel object recognition.

## Introduction

Impaired short-term memory, cognitive symptoms, such as speech difficulties as well as psychiatric symptoms, such as anxiety, agitation, bizarre behavior, delusional thoughts, and hallucinations are major hallmarks in patients with limbic encephalitis, in particular when associated with autoantibodies against N-methyl D-aspartate receptors [NMDARs, (1–3)]. On the molecular level, NMDARs are composed of GluN1 and GluN2 subunits, and it was demonstrated that NMDAR autoantibodies derived from patients' cerebrospinal fluid (CSF) target the GluN1 extracellular domain leading to internalization of the entire NMDAR complex (2, 4–6). Since there is overwhelming evidence that NMDARs are instrumental for memory acquisition and retrieval [reviewed by Nakazawa et al. (7)], it is an attractive hypothesis that autoantibodies targeting NMDARs negatively interfere with NMDAR function and thus impair memory formation directly. Several studies found that both commercial and patient CSF-derived NMDAR antibodies blocked long-term potentiation [LTP, (8–11)] which is a major NMDAR-dependent function regarded as molecular key mechanisms in memory formation (12). Hence, these studies provided strong evidence for the direct pathogenicity of these autoantibodies, and in fact, it is currently believed that autoantibodies raised against neuronal surface proteins, such as transmitter receptors, play an important pathophysiological role in autoimmune encephalitis and thus may be the immediate cause of typical symptomatology (13).

Behavioral studies have demonstrated that spatial memory in rodents heavily depends on intact hippocampal function and activation of NMDARs (7). Importantly, consistent with the LTP data described above, it was observed that hippocampus-dependent spatial memory formation was also impaired in animals treated with NMDAR-antibodies (8, 14, 15). In contrast, non-spatial memory, such as novel object recognition (NOR) also involves NMDAR function (16), but appears to be a crucial function of the perirhinal cortex (17–21). This behavioral task typically consists of a sample phase where the animal freely explores two objects in order to get familiarized, and following a variable delay, one of the two objects is replaced by a novel one (22, 23). In this choice phase, the animal might recall the familiarized object and hence spend more time to explore the novel one (16). Importantly, object recognition was preserved after hippocampal lesions (24–28), and, moreover, only perirhinal cortex, but not the hippocampus was required when the two objects of the sample phase were identical (25, 29). However, CA1-specific ablation of the GluN1 subunit mice prevented object recognition memory (30), and intra-hippocampal infusion of the NMDAR blocker AP5 before acquisition impaired recognition memory after a delay of 3 h (31). Since we aimed to study NOR in anti-NMDAR encephalitis, we first tested CA1-LTP as a characteristic NMDAR-dependent function in our previously established rat model of anti-NMDAR encephalitis that showed impaired Morris water maze memory performance and reduced LTP in the dentate gyrus and CA3 (8, 10). Thus, we hypothesized that CA1-LTP was also compromised in this model.

Recently, it was reported that mice with chronic intracerebroventricular pump infusion of patient-derived NMDAR-encephalitis CSF exhibited impaired spatial memory in the Morris water maze suggesting that hippocampal function was compromised in these animals, while NOR impairment failed to reach statistical significance (14). However, the novelty object recognition was tested 24 h after a single acquisition phase, and hence such a long delay may have been influenced by habituation effects. Moreover, previous reports have shown that perirhinal NMDAR were required for a long delay between acquisition and novelty recognition (32, 33), but their role for shorter delays is less clear (21, 33, 34). Recent reports using a retention phase of 3 h showed a marked phenotype in mice treated with NMDAR-CSF using cerebroventricular infusion (15, 35). We performed a single bolus injection into the hippocampus in order to delimit the potential antibody diffusion and hypothesized that NOR tested a few hours after acquisition might be also affected and, therefore, we performed a methodologically different approach with two sample phases for acquisition in order to allow the rats to habituate and the recognition test a few hours after the second sample phase.

## Materials and Methods

### Cerebrospinal Fluid Sampling

Cerebrospinal fluid (CSF) was collected via lumbar puncture from four anti-NMDAR encephalitis patients (CSF N1-4) and five epilepsy patients (CSF C1-5) with confirmed absence of anti-NMDAR (Table 1). Lumbar puncture was performed either during the diagnostic workup (no immunotherapy at the time of CSF sampling: cases N2, N4 in Table 1) or during the follow-up in a case of high-titer anti-NMDAR encephalitis (case N1, Table 1). Thus, the time from the manifestation of symptoms to the CSF sampling varied between 2 months and 6 years. After collecting the CSF, a sample of 1–2 ml was frozen immediately, and only samples devoid of contamination with erythrocytes were used for our studies. All CSF samples were kept frozen until being used for the experiments. Hence, CSF samples still contained all proteins, but were practically cell-free. The anti-NMDAR antibody titer was determined by end-point-titration of the characteristic NMDAR antibody staining pattern on rat brain through indirect immunohistochemistry done by C.G.B. (36); the NMDAR antibody reactivity was confirmed by a cell-based assay performed by Angela Vincent [Oxford/UK; (37)]. The patients have given their written informed consent to use the CSF samples for scientific purposes.

**Table 1:** Cerebrospinal fluid (CSF) samples.

**CSF**	**Disease, clinical information**	**Sex**	**Age**	**NMDAR-ab titer**	**# of animals**
N1	Epilepsy, anti-NMDAR encephalitis Cognitive problems, seizures and psychosis Prednisolone, cyclophosphamide, plasma exchange	F	25	1:512	5
N2	Epilepsy, anti-NMDAR encephalitis Cognitive problems, psychosis No immunotherapy	M	19	1:32	5
N3	Epilepsy, anti-NMDAR encephalitis	F	19	1:32	8
N4	Epilepsy, anti-NMDAR encephalitis Cognitive problems, psychosis No immunotherapy	F	22	1:64	14
C1	Epilepsy, focal cortical dysplasia 2b	F	31	Negative	6
C2	Post-traumatic epilepsy	M	74	Negative	4
C3	Epilepsy, amygdala tumor	F	42	Negative	8
C4	Post-traumatic epilepsy	M	41	Negative	7
C5	Epilepsy, ganglioglioma	F	16	Negative	7

### Stereotactic Intrahippocampal CSF Injection *in vivo*

**Figure 1:**
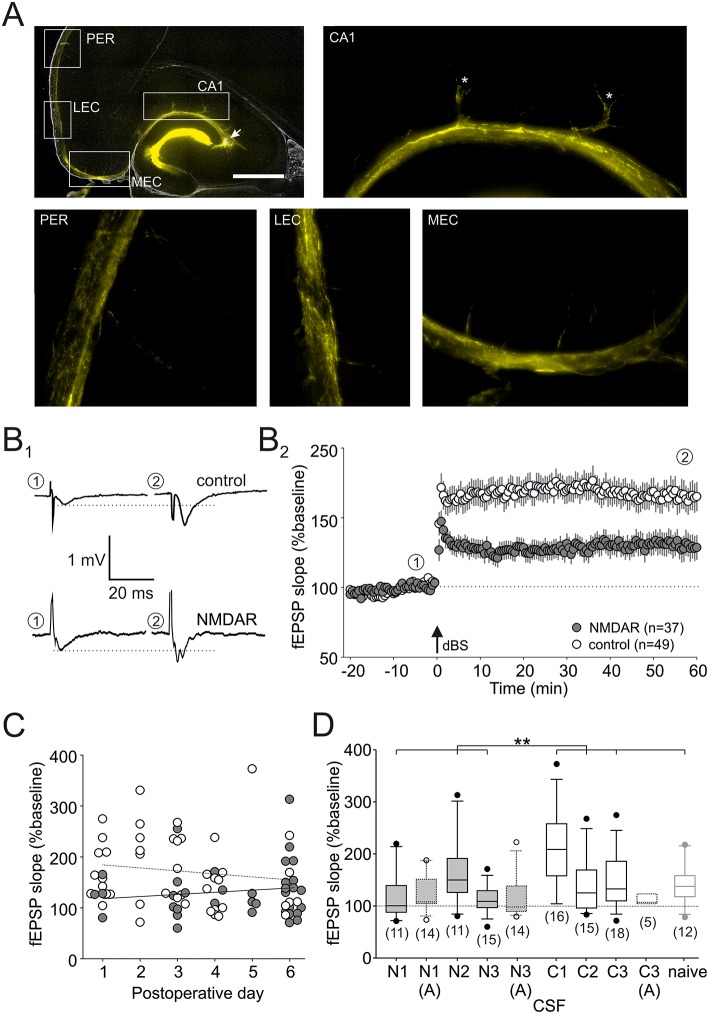
LTP deficit in hippocampal CA1. **(A)** Immunofluorescence micrographs showing the marker dispersion in the hippocampus 1 h after injection into CA3 stratum radiatum (denoted by an arrowhead), magnification 20×. Note that the marker intensely diffuses into the dentate gyrus, but also reaches CA1 and the parahippocampal gyrus. The white boxes indicate the positions of enlarged micrographs (magnification 200×): CA1, Cornu Ammonis 1; MEC, medial entorhinal cortex; LEC, lateral entorhinal cortex; PER, perirhinal cortex. The scale bar indicates 1,000 μm. **(B)** Schaffer collateral–CA1 synaptic long-term potentiation (LTP) is significantly reduced in slices from NMDAR-CSF-treated rats. Representative traces **(B**_**1**_**)** were taken at the time-points indicated in the time course **(B**_**2**_**)**. The arrow indicates the time-point of delta-burst stimulation (dBS). **(C)** There was no significant correlation between the LTP level and the post-operative day in both groups. **(D)** Box plot showing the LTP magnitude of all groups (NMDAR-CSF: N1–3; control-CSF: C1–3; naive) at the end of the experiment (**P < 0.01). Experiments in the presence of the NMDAR blocker D-AP5 (indicated by “A”) are presented with dotted lines.

Stereotactic injection of CSF from patients into both hippocampi *in vivo* was performed as previously described (8, 10). Briefly, 65 female Wistar rats (8–10 weeks old, 190–220 g) were anesthetized with S-ketamine (100 mg/kg i.p.) and xylazine (15 mg/kg i.p.), and mounted on a stereotactic frame (Narishige, Tokyo, Japan). For the injection of native, non-diluted CSF (10 steps of 0.5 μl every 2 min, total of 5 μl for each side), a Hamilton syringe (75N; Hamilton AG, Bonaduz, Switzerland) was inserted into the hippocampus using the following coordinates: 5.2 mm posterior, ±4.3 mm lateral, 4.8 mm deep (relative to bregma). The site of CSF diffusion was predicted from experiments using injection of an immunofluorescent marker dye, cryostat sections of this brain were covered with ProLong® Gold antifade reagent with DAPI (Invitrogen) and evaluated using the Leica DMI6000B microscope and LAS AF software (Figure 1A). After completing the injection, the syringe was left *in situ* for another 2 min to enable CSF diffusion into the hippocampus. After surgery, the rats were given metamizole (100–150 mg/kg) for post-operative pain control and allowed to recover in an atmosphere with enhanced oxygen fraction (4–5 l/min in an 8 l glass vessel). There was a low rate of minor perioperative morbidity (1 case of bleeding) and no severe morbidity or mortality (0/65). All procedures were performed according to national and international guidelines on the ethical use of animals (European Council Directive 86/609/EEC, approval of local authority LALLF M-V/TSD/7221.3-1.1-017/11), and all efforts were made to minimize animal suffering and to reduce the number of animals used.

### Electrophysiological Recordings and LTP Induction

Hippocampal slices were prepared 1–8 days after stereotactic surgery (8, 10). Briefly, rats were decapitated in deep anesthesia with diethyl ether, the brains were rapidly removed and submerged into oxygenated ice-cold dissection solution containing (in mM) 125 NaCl, 26 NaHCO_3_, 3 KCl, 1.25 NaH_2_PO_4_, 0.2 CaCl_2_, 5 MgCl_2_, and 13 D-glucose (95% O_2_, 5% CO_2_; pH 7.4; 306–314 mosmol/kg). Horizontal hippocampal brain slices (400 μm) were cut using a vibratome (Campden Instruments, Loughborough, UK), and slices were then transferred into a holding chamber containing artificial cerebrospinal fluid (ACSF) containing (in mM) 125 NaCl, 26 NaHCO_3_, 3 KCl, 1.25 NaH_2_PO_4_, 2.5 CaCl_2_, 1.3 MgCl_2_, and 13 D-glucose (306–314 mosmol/kg, bubbled with 95% O_2_ and 5% CO_2_ to maintain the pH at 7.4).

LTP was assessed by recording field excitatory post-synaptic potentials (fEPSPs) from CA1 area. The slices were continuously bathed in oxygenated ACSF (flow rate of 2 ml/min, temperature 32 ± 1°C, npi electronic GmbH, Tamm, Germany). For the stimulation of Schaffer collaterals, bipolar stimulating electrodes were fabricated from teflon-insulated platinum wire electrodes (PT-2T, Science Products, Hofheim, Germany). Stimuli were delivered through a stimulus isolator (A365, World Precision Instruments, Sarasota FL, USA) triggered by a Master-8 stimulator (A. M. P. I., Jerusalem, Israel), and LTP was induced by a paradigm consisting of 10 trains of 20 stimuli at 100 Hz (stimulus duration 100 μs, intertrain interval 800 ms, referred to as delta-burst stimulation [dBS]) at double baseline stimulation intensity. Analog recording signals were amplified, filtered at 1 kHz by an EXT-10-2F (npi electronic GmbH, Tamm, Germany), and digitized with a Micro1401 analog-to-digital converter (Cambridge Electronic Design, Cambridge, UK) using Signal 2.16 software (Cambridge Electronic Design, Cambridge, UK). D-(-)-2-amino-5-phosphonopentanoic acid (D-AP5) was purchased from Tocris. All other chemicals used for physiological solutions were purchased from Sigma-Aldrich (Taufkirchen, Germany).

### Analysis of Spontaneous Behavior and Object Recognition

The observation of spontaneous behavior was started 4 or 5 days after stereotactic surgery (referred to as post-op 4.5). Animals were transferred to the behavior analysis room 1 day prior to the observation experiments for habituation to the environment and stayed in this room for the whole behavioral analysis. Room temperature was recorded (20–22°C), and lights were automatically switched on from 06:00 to 18:00 h. The behavioral observation unit was completely separated from the cages in order to prevent optic or acoustic disturbance of the rats that were currently observed. Food and water was given *ad libitum*. All materials that have been in contact with the animal tested were washed with acetic acid thereafter in order to prevent olfactory cues for the next animal.

**Figure 2:**
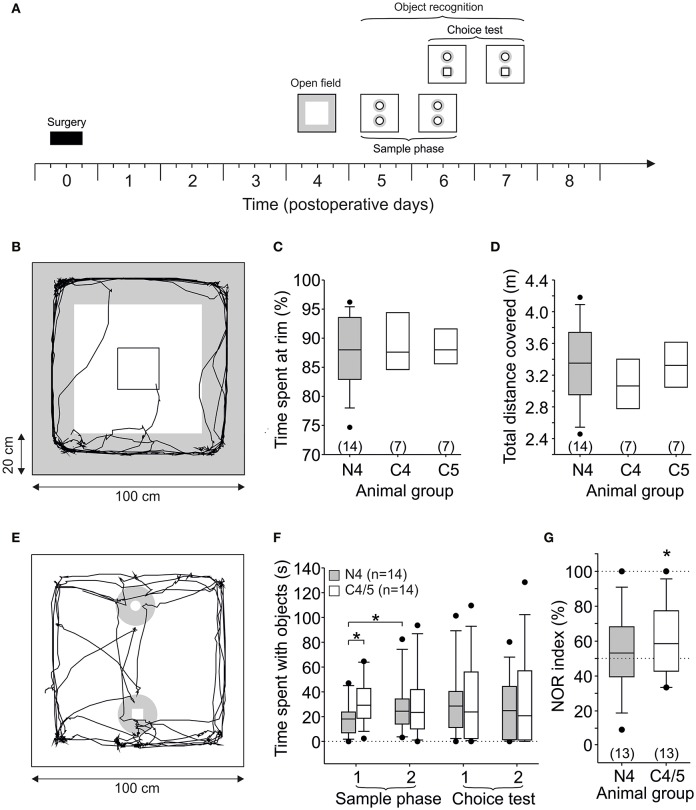
Open field and novel object recognition. **(A)** Time frame of the behavioral experiment. The open field test was performed on post-operative day 4 or 5 (here only shown for animals which started on post-op day 4). Object recognition was tested on three consecutive days. Note that sample phase 2 and choice test 1 are on the same day with a delay of 1.5–3 h. **(B)** Scheme of the arena (100 × 100 cm) with trajectory of a control animal (group C5) inserted within the center which stays at the 20 cm rim for almost the complete period. **(C)** Time spent at the rim (20 cm) of the arena was not different between all three groups. **(D)** Total distance covered during 5 min was also not different between all three groups. **(E)** Scheme of the arena equipped with two objects, and exploration was defined as presence of the animal close to the object (diameter 18 cm, gray zone). **(F)** Time spent with the objects in NMDAR-CSF-treated and control rats during the behavioral experiment. **(G)** NOR index for choice test 1. Note that random presence of the animal at the objects would lead to a NOR index of 50%. The asterisk indicates a significant difference of the observed values against this chance level of 50%. Two animals (one NMDAR-CSF-treated and one control rat) spent no time at both objects during the choice test, these animals were not included for the NOR index calculation (i.e., *n* = 13 instead of 14). (*P < 0.05).

First, we tested spontaneous exploration behavior with the open field test (post-operative day 4.5). Briefly, the rats were placed into the center of a 100 × 100 × 50 cm black polyvinyl chloride box for 5 min, and the trajectories were recorded with a video camera connected to the tracking software EthoVision Color (Noldus, The Netherlands). After the open field, we analyzed NOR. To this end, the rats were placed into the arena as above which was equipped with two identical cylindrical objects (uniform gray color, diameter 4.5 cm, height 7 cm). This sample phase was performed on post-operative day 5 or 6 (referred to as post-op 5.5) and on post-operative day 6 or 7 (referred to as post-op 6.5). Following a delay of 1.5–3 h, and hence still on post-operative day 6.5, the first choice test was performed by replacing one of the cylindrical objects by a cuboid black-and-white colored object (5 cm square, height 5.5 cm, Figure 2A). This choice test was repeated on the following day (post-operative day 7.5). For the analysis of the exploring behavior, we defined a circular area around the object (diameter 18 cm, see light gray zones in Figure 2E) and defined exploration as the time spent within this circle. In addition, we calculated the NOR index as the percentage of time spent at the novel object divided by the total time spent at both objects (i.e., within the diameter of 18 cm). In case the total time spent at both objects during the choice test was zero, these animals were not included for the NOR index calculation (in both choice tests one NMDAR-CSF-treated and one control rat, respectively).

### Statistical Analysis

Data are expressed as mean values ± the standard error of the mean (SEM). For statistical evaluation, data were first tested for normal distribution and equal variance (SigmaStat 3.5). Depending on this normality test, statistical comparisons were performed either using parametric (paired or unpaired *t*-test, ANOVA) or non-parametric tests (Mann-Whitney test or Kruskal-Wallis test) as indicated. The level of significance is indicated by asterisks (^*^P < 0.05, ^**^P < 0.01).

## Results

### CA1 Long-Term Potentiation

In the present study, we aimed to analyze spontaneous exploration behavior in rats that have received a stereotactic intrahippocampal injection of CSF containing NMDAR-antibodies. But first, we confirmed the site of injection using an immunofluorescent marker dye (CA3 stratum radiatum, see arrow in Figure 1A, leftmost upper panel) showing that the injected volume was diffusing along the vessels into the CA1 parenchyma, but also into medial and lateral entorhinal cortex as well as the perirhinal cortex (see enlarged panels in Figure 1A). Since LTP at Schaffer collateral–CA1 synapses induced by dBS is typically NMDAR-dependent (38), we expected impaired LTP as these synapses. Figure 1B_1_ shows representative field excitatory post-synaptic potentials (fEPSPs) recorded in CA1 stratum radiatum upon stimulation of Schaffer collaterals, and on average, the magnitude of LTP in slices from NMDAR-CSF-treated rats (CSF N1-3, titer of 1:512 and 1:32; LTP: 131 ± 11% of baseline, *n* = 37; gray symbols in Figure 1B_2_) was significantly lower than in control-injected rats (CSF C1–3; LTP: 170 ± 10% of baseline, *n* = 49; open symbols in Figure 1B_2_; *P* < 0.01, Mann-Whitney *U*-test, for CSF samples see Table 1). Since we aimed to study explorative behavior within the first week after stereotactic injection, we analyzed whether LTP level was dependent on the time-point after surgery, but found no significant correlation in slices from both control-injected (open symbols in Figure 1C; Pearson correlation coefficient = −0.1453, *n* = 49, *P* > 0.05, *t*-test) and NMDAR-CSF-injected animals (Pearson correlation coefficient = 0.1151, *n* = 37, *P* > 0.05, *t*-test). These results suggest that during the first week the duration of the post-operative period did not affect Schaffer collateral–CA1 LTP magnitude, but demonstrates the impairment of this form of LTP in NMDAR-CSF-injected animals as reported previously for the dentate gyrus and CA1 (8, 10).

While the overall LTP magnitude was significantly lower in slices from NMDAR-CSF-injected animals as compared to control animals, we had to use different CSF samples in these experiments due to the limited availability of patient material. In order to control for cohort effects, we calculated the LTP magnitudes in separate subgroups (NMDAR-CSF: N1–3; control CSF: C1–3, Table 1) as well as in non-operated naive animals (Figure 1D). These analyses revealed no significant differences among NMDAR-CSF-treated tissue subgroups (*P* = 0.096, Kruskal-Wallis test), although there was one subgroup of NMDAR-CSF-treated animals (N2) showing significant LTP when compared to baseline values (indicated by a dotted line in Figure 1D). On the other hand, one control subgroup showed higher LTP magnitudes, but there was also no significant difference among the control subgroups (*P* = 0.033, Kruskal-Wallis test, Dunn's *post-hoc* tests for all comparisons: *P* > 0.05). Hence, one of three NMDAR-CSF subgroups showed significant LTP compared to all of four control subgroups (*P* = 0.053, χ^2^ test). This subgroup analysis demonstrated that Schaffer collateral–CA1 LTP is generally reduced in slices from NMDAR-CSF-injected animals as compared to tissue from control CSF-injected rats without relevant cohort effects. In addition, we found that the overall LTP magnitude of slices from control-CSF-injected animals was not significantly different from the LTP magnitude obtained in non-operated, naive animals (*P* > 0.3, Mann-Whitney *U*-test).

Next, we tested the NMDAR dependence of LTP under our conditions by using the NMDAR inhibitor D-AP5 (50 μM). In both NMDAR-CSF-treated subgroups tested, there was a residual potentiation indicating some NMDAR-independent portion of LTP (N1: 125 ± 9% of baseline, *n* = 14; N3: 119 ± 11% of baseline, *n* = 14; Figure 1D). Importantly, these values were almost identical to the residual potentiation obtained in slices without D-AP5 from these subgroups (N1: 122 ± 14% of baseline, *n* = 14; N3: 113 ± 7% of baseline, *n* = 14). Hence, the residual potentiation following NMDAR-CSF injection was largely attributable to NMDAR-independent LTP. Moreover, there was no significant difference between these two NMDAR-CSF subgroups and control-CSF-injected tissue (CSF C3: 114 ± 4% of baseline, *n* = 5; Figure 1D) suggesting that NMDAR-independent LTP levels were not influenced by NMDAR-CSF.

### Spontaneous Locomotor and Anxiety-Related Behavior

Having shown that stereotactic injection of NMDAR-CSF attenuates LTP in the hippocampal CA1 area for up to 8 days, we asked whether or not spontaneous behavior including NOR is compromised in these animals. To this end, rats were stereotactically injected with either NMDAR-CSF (CSF N4, titer 1:64) or with one of two controls that have been used in an earlier study (CSF C4 and C5; 8). First, the animals were placed for 5 min into an open field in order to observe spontaneous behavior on day 4 or 5 after surgery (referred to as post-op 4.5; Figure 2A). A representative trajectory of spontaneous locomotor activity is depicted in Figure 2B (control animal of group CSF C5) demonstrating that the animal spent most of the time at the rim of the open field (20 cm from the outer wall; indicated in gray). This thigmotactic behavior is characteristic for rodents in a novel environment with open spaces (39, 40), and the average time spent at the rim did not differ between the NMDAR-CSF-treated group (CSF N4: 88 ± 2%, *n* = 14) and two control groups (CSF C4: 89 ± 1%, *n* = 7; C5: 90 ± 2%, *n* = 7; ANOVA; Figure 2C). Likewise, the time spent at the center of the open field was similar among all groups (data not shown). Since thigmotaxis is regarded as anxiety-related behavior, these data do not support an impairment of anxiety-related behavior in our anti-NMDAR encephalitis model, at least in the open field—in contrast to a more aversive environment, the Morris water maze (8). The total distance covered by the animals during 5 min exploration time revealed no significant differences either (CSF N4: 3,336 ± 131 cm, *n* = 14; CSF C4: 3,382 ± 119 cm, *n* = 7; CSF C5: 3,159 ± 155 cm, *n* = 7; ANOVA; Figure 2D) indicating similar motivation to explore the environment. In summary, intrahippocampal injection of NMDAR-CSF with consecutive CA1 NMDAR dysfunction did not change spontaneous locomotor activity or anxiety-related behavior.

### Novel Object Recognition

Following the open field test, we analyzed object recognition in NMDAR-CSF-treated animals. To this end, this observation period of 5 min in the same arena was repeated on three consecutive days (Figure 2A), but the arena was equipped with two identical cylindrical gray objects (sample phase in Figure 2A). Since the animals were habituated to the arena, they approached and explored these objects, and exploring the objects was defined as presence within a circular area (diameter 18 cm, light gray zone in Figure 2E) around each object. Since there were no differences between both control groups (CSF C4 and C5), we pooled the data in this object recognition analysis (referred to as control). During the first sample phase, NMDAR-CSF-treated rats spent significantly less time with these objects (18 ± 4 s, *n* = 14) than controls (31 ± 5 s, *n* = 14, *P* < 0.05, unpaired *t*-test; Figure 2F). When this sample phase was repeated on the subsequent day, NMDAR-CSF-treated and control rats showed equal amounts of time spent at both objects (CSF N4: 29 ± 6 s; CSF C4/5: 30 ± 7 s; Figure 2F). Thus, NMDAR-CSF-treated rats significantly increased the time exploring both objects (*P* < 0.05, paired *t*-test; Figure 2F) suggesting a subliminal degree of anxiety-related behavior which may have been missed in the open field. Hence, during the second sample phase, both experimental groups showed similar levels of motivation, curiosity, and interest toward the objects.

On the same day, but 1.5–3 h later, one object was replaced by a novel one (cuboid, black-and-white, choice test in Figure 2A), and we asked whether the animals would now explore the novel objects defined as being close to the object. In this choice test 1, NMDAR-CSF-treated, and control rats spent similar time at the novel object (CSF N4: 17 ± 4 s *n* = 14; CSF C4/5: 19 ± 5 s, *n* = 14; *P* > 0.7, unpaired *t*-test; Figure 2F). Novelty recognition was then assessed by calculating the NOR index as ratio between the time spent at the novel object divided by the time spent at both objects in this choice test. The direct comparison of the NOR index between both experimental groups failed to reach statistical significance (CSF C4/5: 61 ± 6%, *n* = 13; CSF N4: 52 ± 5%, *n* = 13, *P* = 0.095, unpaired *t*-test). The *post-hoc* power analysis revealed that the effect size was acceptable (*d* = 0.21), but the power was still rather low (1–β = 0.28) probably attributing the failure of detecting statistical significance to the small sample size. However, the NOR index is expected to be higher than 50% when novelty is recognized, and significant NOR was in fact observed in control animals spending more time with the novel object than expected by chance (*P* < 0.05, paired *t*-test; Figure 2G), which was not the case in the NMDAR-CSF-treated group (*P* > 0.3, paired *t*-test).

In order to test for habituation, we repeated this choice test on the following day and obtained almost equal mean values for both groups (control: 52 ± 8%, *n* = 13, *P* > 0.4; NMDAR-CSF: 43 ± 8%, *n* = 13, *P* > 0.2) indicating that control animals had habituated to the novel object. Taken together, NMDAR-CSF-treated animals showed significantly suppressed CA1 LTP, and, moreover, impaired NOR. These data are consistent with the idea that proper NMDAR function is involved in rodent recognition memory.

## Discussion

In this study, we aimed to explore spontaneous behavior and recognition memory in animals after stereotactic intrahippocampal injection of CSF from NMDAR encephalitis patients (NMDAR-CSF). Recent data from Li et al. (14) also using NMDAR-CSF suggested that hippocampal NMDARs were crucial for Morris water maze performance, but possibly not for NOR. Previously, we found impaired dentate gyrus LTP and Morris water maze performance in rats after bolus injection with NMDAR-CSF (8).

### NMDAR Dysfunction in the Hippocampal CA1 Subfield

Here, we first aimed to demonstrate the unambiguous effect of NMDAR-CSF in our model and obtained LTP data from the hippocampal CA1 area. These experiments showed a significantly lower magnitude of LTP in NMDAR-CSF-treated tissue indicating that NMDAR function was compromised in the CA1 field of these animals. Impaired NMDAR-dependent LTP in tissue treated with NMDAR-antibodies is consistent with previous data from different research groups (8–11) and underlines the overwhelming role of NMDARs in CA1-LTP (41). Moreover, the present study has shown that residual potentiation in NMDAR-CSF-treated animals was identical to LTP obtained under pharmacological NMDAR inhibition. These findings suggest that autoantibodies against NMDA receptors might have blocked almost all the NMDAR-dependent LTP and NMDAR-independent mechanisms of plasticity remained intact. Interestingly, residual levels of LTP were also observed in previous reports using both patient CSF and commercial GluN1-antibodies in the CA1 and CA3 area (8–10), but not in the dentate gyrus (8), which might point to NMDAR-independent mechanisms being particularly present in CA1 and CA3. Based on the literature (38, 42, 43), CA1-LTP induced by burst stimulation in the delta range is predominantly mediated by NMDA receptors, but Ca^2+^ entry through voltage-dependent Ca^2+^ channels cannot be excluded and thus may play a major role in this NMDAR-independent LTP. Lastly, it is conceivable that the residual LTP in NMDAR-CSF-treated tissue could be due to differential NMDAR sensitivity depending on their subunit composition. The epitope of NMDAR-antibodies derived from patients is known to be the extracellular domain of the GluN1-subunit (2, 4–6).

With respect to post-operative delay, we did not observe significant impact of the post-operative day on the LTP levels in both NMDAR-CSF-treated and control animals. Rather, we found that LTP in NMDAR-CSF-treated tissue remained significantly smaller for up to a week. This prolonged effect of patient-derived NMDAR-antibodies in the hippocampal CA1 field could also be observed in the dentate gyrus (8), and was a prerequisite for our behavioral studies. Another issue might be the female sex of our animals, since the rat menstrual cycle is about 4–5 days (44) and may influence the LTP magnitude. However, our time span of experiments completely covered the full menstrual cycle, and both NMDAR-CSF-treated and control-operated rats showed similar variance arguing against a major impact of the menstrual cycle in the LTP magnitude.

We have tested three different NMDAR-CSF samples and four control groups using control CSF from patients with epilepsy, but with proved absence of NMDAR-antibodies as well as non-operated, naive animals. Although there was no significant difference among the three NMDAR-CSF subgroups and among the four control subgroups, one NMDAR-CSF-treated subgroup exhibited significant LTP, indistinguishable from controls (CSF N2) and one control-CSF subgroup appeared to have supernormal values of LTP (CSF C1). These findings emphasize that CSF samples from individual patients with different epitope-targeted NMDAR-antibodies might substantially differ in their capacity to block NMDAR-dependent LTP. Importantly, LTP in animals treated with an extraordinary high titer anti-NMDAR-CSF (CSF N1, 1:512) was not significantly different from LTP in animals following injection of CSF N3 (titer of 1:32). Although antibody titers may not directly translate into antibody concentrations, LTP in the N2-subgroup (titer of 1:32) even tended to be of higher magnitude than LTP of this high-titer N1 subgroup. These data implicate that the titer itself does not predict the effect of NMDAR inhibition, in particular when comparing high-titer vs. low-titer patients. While there is a good correlation between the NMDAR-antibody titer and the symptoms in a given patient (45), our data indicate that the inter-individual correlation between the titer and the clinical picture is rather loose. A potential explanation is that the antibody concentration does not necessarily correlate with the titer in blood or CSF (46), e.g., because the blood-brain or CSF-brain crossing may underlie different restrictions (47).

### Spontaneous Behavior and Object Recognition in NMDAR-CSF-treated Animals

The behavioral tests started with the observation of spontaneous explorative behavior in the open field. Here, the total distance covered during the observation period did not differ between the experimental groups indicating that spontaneous locomotor activity was entirely normal—consistent with previous reports (8, 14). Moreover, we were also unable to detect significant group differences in the time spent at the rim or at the center, markers of anxiety-related behavior. Interestingly, Li et al. (14) observed that NMDAR-CSF-treated mice spent less time at the center of the open field, but this effect turned out to be not statistically significant. However, in our previous study (8), we found significantly increased thigmotaxis in the Morris water maze. Therefore, we assume that anxiety-related behavior might be increased in NMDAR-CSF-treated animals, but depending on the aversiveness of the environment. In this sense, the open field in the present study was rather unaversive.

The major aim of this study was to explore recognition memory in NMDAR-CSF-treated rats using the NOR task. This test is based on the natural behavior of rodents to explore novelty and does not require extensive training (22, 23). In the present study, we detected significant differences in the time spent at the objects during the first sample phase between NMDAR-CSF-treated animals and controls, but equal levels of exploration during the second sample phase 1 day later. These results indicate intact motivation to explore the environment at least during this second sample phase, but we cannot exclude that reduced recognition memory during the choice test was in part influenced by this difference in the first sample phase. Nonetheless, the NOR index revealed a significant capability of control-injected rats to distinguish between the familiar and the novel object, which could not be observed in NMDAR-CSF-treated animals. This is an important finding which, on the one hand, is consistent with the idea that proper NMDAR function is involved in rodent recognition memory, and on the other hand, helps disentangle the specific roles of hippocampal structures in recognition memory. While there is a large body of evidence coming from hippocampal lesion studies that consistently showed retained object recognition (24–29), a few functional studies using either pharmacological NMDAR inhibition or CA1-specific ablation of the GluN1 subunit clearly demonstrated the involvement of hippocampal NMDARs in object recognition memory (30, 31). Anatomically, there are extensive monosynaptic and reciprocal connections between the perirhinal cortex and the hippocampus, in particular the CA1 subfield and the subiculum (48, 49), but not the dentate gyrus (50). In addition, a number of studies have suggested that NMDARs located within the perirhinal cortex are critically involved in recognition memory (21, 34, 51). Hence, it is likely that NMDAR-CSF diffusion to the perirhinal cortex as evidenced by the immunofluorescent marker diffusion has led to the lack of novelty recognition in these animals.

However, there are some arguments that have questioned this view. Firstly, perirhinal NMDAR dependence was required for a rather long delay between acquisition and novelty recognition of up to 24 h (32, 33), while recognition memory was unaffected after a short delay of 1 h, even though NMDARs in the perirhinal cortex were already blocked during acquisition (21, 33, 34). Here, we pre-applied the NMDAR-CSF more than 5 days prior to the acquisition and had a rather short delay of 1.5–3 h, thus reducing the possibility of NMDAR dysfunction in the perirhinal cortex. Secondly, intra-perirhinal inhibition of GluN2A and GluN2B antagonists restrained object recognition only in case of co-application, but none of them produced any impairment when administered solely (34). Thirdly, NOR has also been analyzed using the model of chronic intracerebroventricular NMDAR-CSF administration (14). Using this approach, perirhinal diffusion of NMDAR-CSF should be assumed. The authors, however, found only a trend toward higher novelty discrimination in control-CSF-treated mice, which failed to reach statistical significance—partly due to the sample size of eight mice per group. In our hands, significant novelty recognition was present in controls and absent in NMDAR-CSF-treated animals, but we were unable to detect significant group differences using sample sizes of 13 animals, respectively. Therefore, it is possible that this negative finding was due to the sample size.

There is another issue that needs to be addressed. It is possible that the injected CSF samples may contain further biologically active ingredients other than autoantibodies and, in addition, that these additional compounds may have effects on behavioral experiments rather than on LTP. However, the NMDAR-specificity has been shown in a recent study comparing NMDAR-dependent and NMDAR-independent LTP in CA3 (10). Thus, we suggest that at least the initial step in object recognition is NMDAR-dependent. In summary, we conclude that there is a rather mild phenotype in object recognition in NMDAR-CSF-treated animals, but this may suggest that NMDARs in the CA1 subfield are involved in rodent NOR, while the specific attributable impact of these receptors is less clear. Future studies will address this particular issue and—in addition—could use CSF from a given patient before and after immunotherapy to serve as the own control in order to enable a paired comparison.

In conclusion, we demonstrated that intrahippocampal administration of NMDAR-CSF impaired CA1 NMDAR function in the *in vitro* slice preparation and produced a lack of novelty recognition in the behavioral analysis. These findings will help shed more light on the pathophysiology of anti-NMDAR encephalitis and adds to the knowledge that autoantibodies against NMDARs are pathogenic in nature.

## Ethics Statement

The patients have given their informed consent to use the CSF samples for scientific purposes. All procedures were performed according to national and international guidelines on the ethical use of animals (European Council Directive 86/609/EEC, approval of local authority LALLF M-V/TSD/7221.3-1.1-017/11), and all efforts were made to minimize animal suffering and to reduce the number of animals used.

## Author Contributions

MK, TR, RB, KP, and TS performed experiments. CB, RK, and TK contributed conception and design of the study. MK, TR, and RB organized the database. MK, TR, RB, and TK performed the statistical analysis. TK wrote the first draft of the manuscript. CB and RK wrote sections of the manuscript. MK, TR, and RB contributed to manuscript preparation. All authors contributed to manuscript revision, read, and approved the final version of this manuscript for submission.

### Conflict of Interest Statement

CB gave scientific advice to UCB (Monheim, Germany) and obtained honoraria for speaking engagements from Eisai (Frankfurt, Germany), UCB (Monheim, Germany), Desitin (Hamburg, Germany), Biogen (Ismaning, Germany), and Euroimmun (Lübeck, Germany). He received research support from Deutsche Forschungsgemeinschaft (Bonn, Germany), Gerd-Altenhof-Stiftung (Deutsches Stiftungs-Zentrum, Essen, Germany), Diamed (Köln, Germany), and Fresenius Medical Care (Bad Homburg, Germany). He is a consultant to the Laboratory Krone, Bad Salzuflen, Germany, regarding neural antibodies, and therapeutic drug monitoring for antiepileptic drugs. The remaining authors declare that the research was conducted in the absence of any commercial or financial relationships that could be construed as a potential conflict of interest. The reviewer AV and handling Editor declared their shared affiliation at the time of review.
